# Counseling (ro)bot as a use case for 5G/6G

**DOI:** 10.1007/s40747-022-00664-2

**Published:** 2022-03-30

**Authors:** Yoshio Taniguchi, Yukino Ikegami, Hiroshi Fujikawa, Yogesh Pathare, Andrea Kutics, Banzi Massimo, Marco Anisetti, Ernesto Damiani, Yoshitaka Sakurai, Setsuo Tsuruta

**Affiliations:** 1 Hitachi Industry & Control Solutions Ltd, Tokyo, Japan; 2 IO inc., Tokyo, Japan; 3 Maruka robotics inc., Osaka, Japan; 4 SYBICA inc., Burlington, Canada; 5 International Christian University, Mitaka, Japan; 6 Telecom Italia, Rome, Italy; 7 Università degli Studi di Milano, Milan, Italy; 8 Meiji University, Tokyo, Japan

**Keywords:** 5G network, Artificial intelligence, Natural language processing, Human–computer interaction (HCI)

## Abstract

This paper presents a counseling (ro)bot called Visual Counseling Agent (VICA) which focuses on remote mental healthcare. It is an agent system leveraging artificial intelligence (AI) to aid mentally distressed persons through speech conversation. The system terminals are connected to servers by the Internet exploiting Cloud-nativeness, so that anyone who has any type of terminal can use it from anywhere. Despite a promising voice communication interface, VICA shows limitations in conversation continuity on conventional 4G networks. Concretely, the use of the current 4G networks produces word dropping, delayed response, and the occasional connection failure. The objective of this paper is to mitigate these issues by leveraging a 5G/6G slice inclusive of mobile/multiple edge computing (MEC). First, we propose and partly implement the enhanced and advanced version of VICA. Servers of enhanced versions collaborate to increase speech recognition reliability. Although it significantly increases generated data volume, the advanced version enables a recognition of the facial expressions to greatly enhance counseling quality. Then, we propose a quality assurance mechanism using multiple levels of catalog, as well as 5G/6G slice inclusive of MEC, and conduct experiments to uncover issues related to the 4G. Results indicate that the number of speech recognition errors in Internet Cloud is more than twofold compared to edge computing, implying that quality assurance using 5G/6G in conjunction with VICA Counseling (ro)bot has higher efficiency.

## Introduction

5G networks have made significant progress towards ultra-reliable low-latency high-speed communication as well as innovative automatic network operations. 6G networks are starting to become defined. However, Dan Hays, principal at PwC’s Strategy & division^1^ says “At this point, 6G is little more than a concept. While it is relatively easy to understand the concept of a next generation of cellular technology beyond 5G, the specific objectives, technologies, and performance that 6G might offer are still very much unknown.” Still more, he says “There are many examples of 5G falling short of its promise with respect to performance, ubiquitous coverage, and use cases^2^. Deployments of fully-capable 5G networks in rural areas are nowhere to be seen, and the fully virtualized capabilities of stand-alone 5G networks are largely absent from most. 6G may well be the communications industry’s opportunity to close these gaps, even if it comes a decade later than expected.” To sum up, 5G falls short of its promise such as network slicing, namely, virtualized capabilities. 6G, currently little more than a concept, could be an opportunity to solve 5G problems including the use cases.

Meanwhile, cognitive computing currently breaks the boundary between neuroscience and computer science. It paves the way for machines to have reasoning abilities analogous to humans. Such ability is called artificial intelligence (AI). However, even at this point, AI is mostly still struggling towards getting out of toy use to really succeed. There is no reported practically useful voice conversation AI (ro) bot. Indeed, voice command systems or voice text translation systems including contextually limited guidance robots [1] are in trial use. However, full-fledged voice conversation systems are still a kind of extras or free add-on to larger systems commercially. Namely, even Siri and Alexa are not mainly used as keyboard or pointing devices for command input.

As to cognitive conversation AI for mental health care, we have developed the following:

1. Context REspectful Counseling Agent (CRECA); a text-based system developed as a baseline system. Owing to maintaining the context during the conversation, CRECA was more than double of ELIZA in the average length of interchanges/conversation, a.k.a. continuation [2, 3].
2. AI counseling agent called VICA (VIsual Counseling Agent) [4].
3. Robot (version of) VICA, namely, VICA’s IoT (Internet of Things oriented robot [5]).

These agent systems help mentally distressed or anxious persons by conversation or interchanges. The components are connected through the Internet and are basically cloud-native, meaning that they can be used from anywhere, by anyone, and with any type or model of terminals. Terminals can either be a software robot (bot) or a physical robot. Counseling/consultancy is a kind of mental operation to mitigate the emotional distress or anxiety of clients. Meanwhile, there are many clients (> 2 million in Japan) who suffer from such disorders as PSTD (Post Traumatic Stress Disorder), disasters, terrorist attacks, pandemics like Corona, or worldwide flu (influenza). Persons with just more simple anxiety or worry are still great in number. Compared with many such patients, there are few human counselors and few hospitals as well as limited telephone capacity. Availability of human counselors/consultants can be significantly low, especially in an emergency such as COVID-19 where possibly over a thousand people may want to consult at once. Thus, AI counselors/consultancy become necessary.

However, on conventional networks such as 4G, VICA has serious problems of conversation discontinuation (short continuation) due to speech recognition quality. Especially in an emergency, the surge in demand easily expects to cause significant discontinuation problems due to the parallel use of VICA. The objective of this paper is a clarification of the method to reduce these problems of short continuation towards Proof of Concept (PoC) of VICA as a 5G use case. This goal is approached as follows: (1) analyzation of speech recognition quality problems, (2) recognition quality enhancement using multiple kinds of speech analyzers, (3) advanced way for continuing conversation using multiple sensors, (4) keeping continuity through multiple catalogue-based assurances of network connection and performance, and (5) estimation of the assurance effect.

This paper is organized as follows. Section 2 overviews our problem statement detail and its solution approach. “VICA: mental care counseling AI application” describes an existing VICA developed for virtual/remote mental healthcare application and its problems and requirements as a 5G use case. “Basic solution to VICA as 5G/6G medical use case” introduces a catalogue-based quality assurance mechanism to solve the problems by fulfilling the requirements on 5G/6G. “Enhanced/advanced VICA as 5G/6G medical use case PoC” proposes the enhanced/advanced version of VICA to make “sensitive listening” more reliable. Then, it describes the experimental method to prove the effectiveness of future 5G/6G networks to VICA. “Evaluation results” evaluates the effects of the experimental results on current networks and by questionnaires about the quality perceived by users. “Related work and comparative analysis” outlines related works, providing the comparison. “Conclusion” concludes the paper.

## Problem statement

In the following, we detail the issues we identified and address our solution/estimation approach.

*Analysis of speech recognition quality problem* Different from the surgical robots, counseling (ro)bots should avoid intrusive operations. This is because such intrusive operations may easily hurt people who are very sensitive due to emotional distress. Instead, our counseling system uses “sensitive (or active) listening” skills with its related methodology for showing congruence, empathy, and unconditional positive regards advocated by Rogers [6, 7]. This is widely disseminated (around 30% used in the United States) and validated by counselors or therapists. VICA realizes these well-known counseling skills and methodology. It promotes conversation or confession (a well-known Christian technique [8]) with digging but with unconditional positive regards, etc. towards self-awareness by the distressed persons themselves. To improve such counseling effect for real use, VICA has implemented a voice speech interface. Due to voice conversation, VICA has become largely different in practical usability from very toyish text-based counseling AI [9], since people do not counsel ordinarily by text communication. Furthermore, VICA uses the best level of cloud-native speech recognizer such as Google speech-to-text cloud-based API (Application Programming Interface). Its high-precision speech recognition capability is especially necessary for our AI counseling to realize the above-mentioned “sensitive listening” skills to promote conversation.

However, in current 4G networks, the speech recognition function for VICA’s voice communication has problems in recognition accuracy/precision [10] as well as its response quality including response time and continuity. These issues need to be addressed for achieving conversation-based practical AI counseling. For this purpose, VICA uses high-performance and scalable cloud-native computing or SaaS (Software as a Service) such as Google speech-to-text cloud service. Indeed, this speech recognition service has the world’s highest level of performance. However, it has generally produced one error every one or two sentences ^4^. Furthermore, it sometimes takes several seconds in response time for a VICA agent to respond. These can cause inevitable difficulty in the conversation for practical AI counseling of our type. In the worst case, the connection/task falls down and the conversation (i.e., counseling) is forced to stop. These are problems of current VICA using voice communication. On the contrary, CRECA (which uses text to communicate) does not have such problems. Even just speech recognition has problems in accuracy and response continuity/time for practical use of AI counselors. Speech-to-text translation errors happen in less than every 10 sentences. It sometimes takes several seconds (sometimes more than 10 s) in response time. It also causes communication errors or even disconnection in a dialogue that is a prerequisite for counseling by VICA. Users become irritated and stop the conversation due to such response problems (disconnection in the worst case) or low quality of speech recognition. It happens even in spite of using the world’s (as of today) highest level of cloud-native speech recognition servers such as Google speech-to-text cloud. VICA terminals need ultra-reliable connectivity to cloud-native high-performance and scalable speech recognition servers.

Furthermore, in emergencies such as pandemics, it can be expected that extraordinarily many people will use counseling/consulting AI systems at the same time. It is accompanied by general usage of network resources for other Web applications including video games, Web meetings, YouTube, etc. For example, the number of persons is expected to be around 70K, while in super mega-cities possibly more than 100k persons [2, 4]. In case of such an emergency, data rate (bandwidth, transmission speed) also becomes a problem, besides the already mentioned network latency problem. High-speed network connectivity should be dynamically supported for up to 70K persons’ sudden concurrent use, though even such number is nothing more than 0.5% of Tokyo citizens. It would become impossible for counseling or consultancy to continue the conversation under these serious conditions. Quick automatic network (slice) operation is necessary, so that distressed people can continue the conversation with VICA under such conditions. At least disconnection should be avoided. Currently, such (network slice) operation is usually done manually, even on software-defined networks. It takes more than 10 min in the worst case. More seriously, VICA terminals have to connect with not only the VICA counseling servers but also cloud-native AI speech recognition servers even on such emergencies.

*Problem of speech recognition quality assurance* To realize more reliability in our “sensitive listening” type counseling, the enhanced version of VICA “(enhanced VICA)” is devised. Servers and clients collaborate for speech recognition error recovery, towards less than one word per around every 20 sentences. For speech recognition quality assurance, recognition/translation error is checked at enhanced VICA servers. At enhanced VICA servers, text level recovery is done using various kinds of n-gram methods such as backward and forward with majority decision [11]. If errors are still suspected, terminals send voice input to speech recognizers again. These are repeated, while errors are suspected. Text transmission latency among servers and clients (if connected over inter-carrier), as well as the processing ability of enhanced VICA servers, are problems to be addressed. Transmission problems are caused in edge, in inter-carrier/Internet, and in cloud centers especially accessed concurrently in case of an emergency. Here, the edge includes radio and carrier backbone (core networks) from access point to providers. Although in text, low-latency connectivity between VICA terminals and servers is required for such recognition error recovery towards less than one-word error per tens of sentences. Besides text data transmission between counseling terminals and servers, voice data should be sent to the recognition cloud servers many times. The total amount of transmitted data becomes larger. It becomes a surge during emergencies. Therefore, enhanced VICA awaits 5G/6G and the agile slicing operation.

*Quality assurance by multi-sensor fusion and its problem* Of course, not all communication is oral in “sensitive listening”. Clients do not always express their emotions in their speech. Advanced version of VICA aiming at a higher quality of “sensitive listening” should communicate with clients not just through voice but also through other kinds of sensors. These sensors recognize the facial expression, heart rate, blood pressure, oxygen level, and so on. Clients’ emotion is recognized not only by speech text but also by sensor signals. These signals are finally translated into text expressing the emotion. This multiple sensor information is obtained by servers in clouds or SaaS. Advanced VICA is proposed to use multi-sensor, especially image sensor fusion AI, so that it can obtain the much higher accuracy. Owing to such fused multiple sensor information, advanced VICA will more accurately know the emotion of clients. This enables the long continuation of counseling conversation to give the emotionally distressed persons a deep awareness. “Sensitive listening” further requires recognition of body language such as facial expression. AI recognition cloud servers of body language such as facial expressions are used for advanced VICA to more reliably detect clients’ emotions or emotional changes on their talk for quality self-awareness. However, for such quality/accurate emotion recognition, it is needed to transmit a large amount of sensor data such as video feeds with less than tens of milliseconds. Thus, advanced VICA expects 5G/6G and the speedy slicing to realize powerful assurance operation inclusive of MEC (Mobile/Multiple Edge Computing) for image recognition.

*Keeping continuity through multiple catalogue-based assurance* To cope with these problems, we introduce a multiple catalogue-based quality assurance mechanisms towards PoC of VICA as a 5G/6G use case.

It is expected to provide optimal cost-performance network quality assurance using 5G/6G and slice inclusive of MEC and Internet cloud. It is necessary for assuring the quality of AI counseling (active listening) by reliable speech recognition, etc. as follows: The proposed service catalogue has multiple levels of the menu according to an emergency degree. For instance, these levels consist of ordinary, prioritized, and (super) highly prioritized levels. The emergency level or a menu selected by users is set as a basic requirement to fulfill network resources including the cloud. The ordinary level does not always assure the recognition quality necessary for counseling continuation, but does just best effort with low price. On the contrary, the prioritized level assures quality in ordinary congestion. The (Super) highly prioritized level assures the contracted service quality even (ultra-) emergencies. According to the service catalogue, base requirements are initially set in each VICA terminal. They are sent to VICA servers. During counseling, VICA clients’ terminals monitor the response time and send it to VICA servers. VICA servers send the base requirements as well as the monitoring results to service providers. It is done through network operation API^3^. Initially, service providers fulfill the base requirement. Then, it assures the service level by network slice operation based on monitoring results. The slice control uses resources such as leased lines, MEC, and so on.

*Estimation of the assurance effect* The problems 1–3 on conventional networks and its solution approach are estimated towards PoC of VICA as a 5G use case as follows:

- Networks of 5G (/6G) with slicing are considered necessary for VICA for even just voice interface (speech recognition). They are very necessary for the advanced VICA of video interface. Experiments are done to prove this towards the PoC of VICA as a 5G use case. The experimental results are analyzed to unveil the problems of conventional networks in our counseling agent VICA. Namely, in conventional networks such as 4G, performance degradation or even disconnection problems happen sometimes. The results/delays are measured and analyzed digitally to clarify the problems. They are beginning with just voice interface level.
- For instance, speech recognition error rate is compared between Internet cloud and edge computing. The result shows the effectiveness of the proposed quality assurance mechanism based on multiple catalogues using a 5G/6G slice inclusive of MEC.

## VICA: mental care counseling AI application

In this section, we describe VICA in greater detail with its problem/requirement for 5G/6G. Later, Sect. 5 proposes VICA enhanced/advanced version that is quality but just partly implemented. This includes both the original (basic) version as well as the enhanced future version.

### Developed AI counselor VICA (basic version)

We proposed and developed a software robot (bot) called VICA: VIsual Counseling Agent [4]. It is a distributed AI (Artificial Intelligent) application for medical services such as mental healthcare to assist emotionally distressed persons. It does a kind of remote mental operation, although not physical. To cope with emergencies such as pandemics, the original system that we call VICA (basic) is gradually extended in consultancy function when questioned for anxiety reduction. It is also going to recognize the emotion, body language, or physical condition of clients (distressed persons) from images of facial expressions and other sensors such as body temperature, etc. This extended VICA is called VICA advanced. In this section, VICA basic is explained as its foundation.

**Fig. 1:**
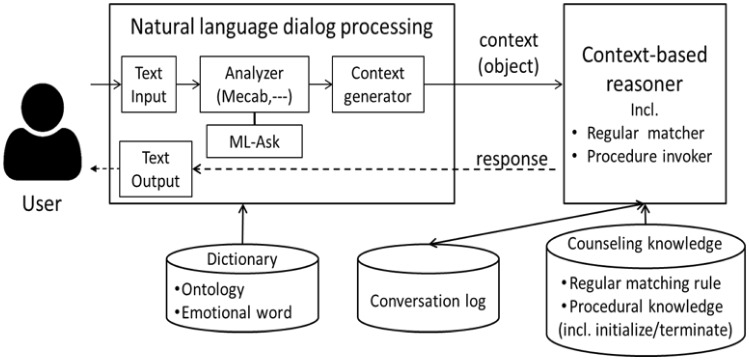
CRECA architecture. *Mecab* Morphological Analyzer (Japanese tokenizer), and *ML-ASK* System for Affect Analysis of Textual Input in Japanese

VICA uses CRECA [2] as a baseline system. CRECA is a text-based system composed as an AI counseling server of VICA. The architecture of CRECA is shown in Fig. 1. Terms and their structure are extracted by standard Natural Language Processing techniques with a dictionary for emotional words and then saved in context objects. The agent creates its dialogue responses using information stored in current context objects and its counseling knowledge database (DB).

CRECA’s natural *language dialogue processing module* consists of (1) an initialization and termination module that functions as an interface between humans and the agent, (2) a dialogue text analysis module, and (3) a dialogue text input /output module. When the CA (Counseling Agent) is launched, the initialization/termination module initializes the context objects and generates the dialogue-starting messages. At the end of the interaction, it saves all relevant information (including the conversation log) and generates the dialogue-end messages.

CRECA uses not only ELIZA-style mirroring but also generates its own responses for digging [5]. Furthermore, it periodically attempts to summarize the clients’ previous utterances considering their emotional impact and chronological order. Asking about the quality of summarization, the agent can check how accurate the listening has been. This also helps clients in seeing their own distortions or reviewing their thinking. Finally, the agent prevents the dialogue from drifting away from the problem even when the client’s emotional state has changed. This preserves the client’s trust in CRECA and keeps the dialogue open.

Sentence patterns used in this phase include standard problem-digging phrases (e.g., “please tell me more” or “give me more details”). Such digging responses give the client a chance for self-reflection.

Different from CRECA, VICA has a voice (speech) and visual interface as its input/output terminals or clients of client–server systems. VICA (or more concretely VICA’s CRECA) is starting to have the function of consultancy or answering to reduce clients’ anxiety. Anyway, it is important for CRECA, and therefore VICA, to continue the conversation as well as recognize a client’s emotional state. Besides the client’s emotional state recognition error, the error in speech recognition is a significant problem.

**Fig. 2:**
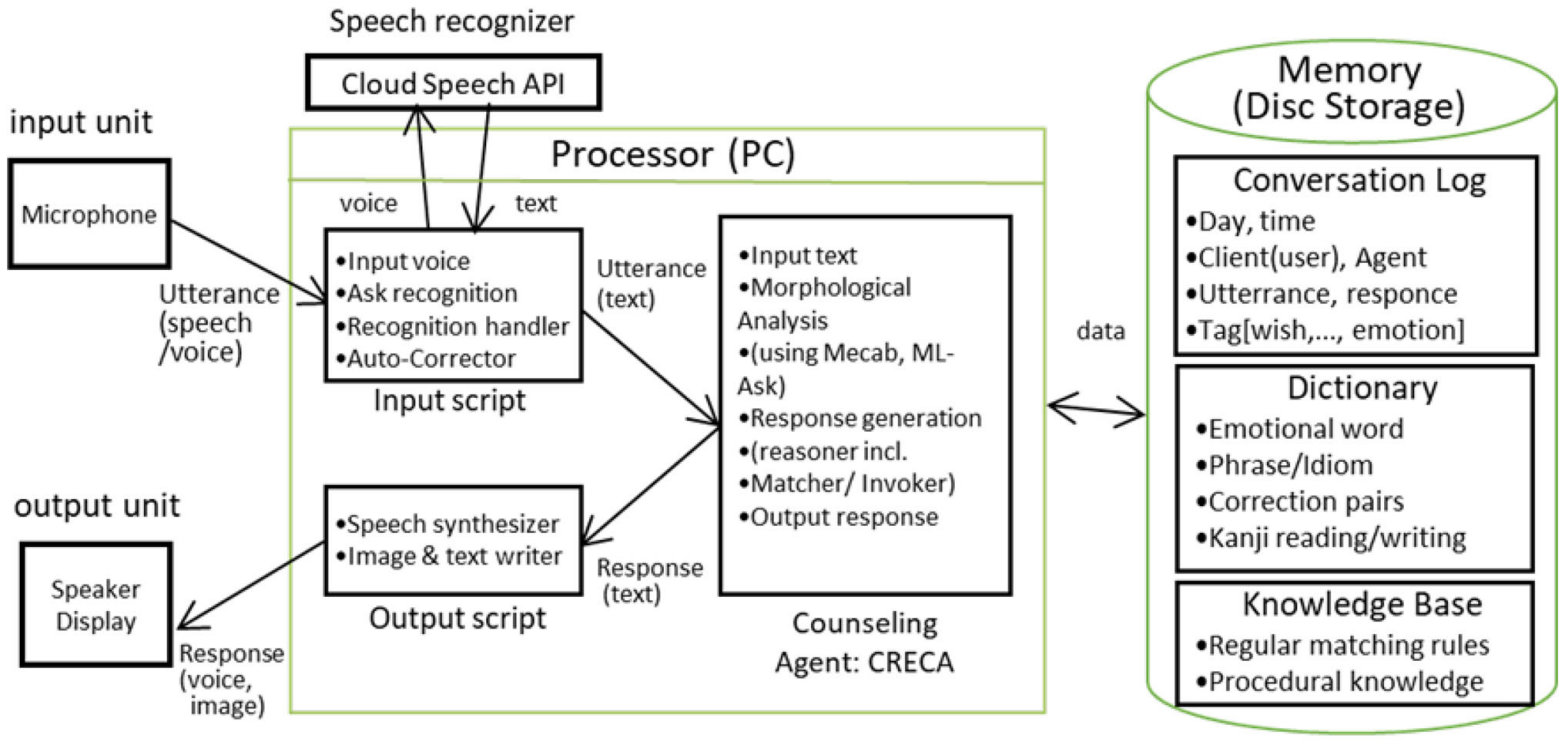
Module structure for VICA’s processing

VICA’s processing flow is as follows:

1. Input of the clients’ voice data
2. Ask speech recognition using speech recognizers such as Cloud Speech API in Fig. 2 to produce texts out of user utterances (client’s voice data) and receive the recognized texts to hand them to CRECA by speech recognition handler as shown in the input script box of 2.
3. Automatic correction of speech recognition results by our interactive software tool works as follows:
  - Activate Google speech recognizer again in case an error code indicating that “insufficient precision” is returned.
  - Execute automatic correction of the speech recognition results, using word/phrase recognition error correction dictionary dynamically improved by our interactive software tool.
4. Display the speech recognition result on a window located above the counselor’s avatar. If the users think it is different from their input (specifically because they spoke in a noisy environment or they could not speak smoothly), they can delete the previous speech input and try again.
5. Generation and logging of the response by CRECA’s context-based reasoning engine that invokes regular matching rules and accompanied procedures generating such as tag questions, based on context, state, or situation:
  - The input text which was handed is taken in.
  - Morphological analysis of the grammatical sentence structure.
  - Generation of response and logs using regular matching rules and accompanying procedures for paraphrasing (including tag questions, personal pronoun/verb conversion) and digging prompts.
6. Generation of response and its logging by CRECA’s extended engine that invokes procedural knowledge based on context (state/situation):
  - Generate a reply confirming emotional changes if recognized by comparing current emotional words with previous ones in the conversation. Here, emotional words can be recognized using an emotional word dictionary and emotional change can be detected using ML-ASK: System for affect analysis of textual input in Japanese [12].
  - If improvement or change of feeling (emotion) is detected, add a corresponding remark or confirmation to the reply and log.
  - If a desire (wish) is expressed, it is added along with the related obstacle, and the resultant feeling/emotion is added to the reply as well as to the log with the tags [wish], [obstacle], and [resultant emotion]. Desires (wishes), obstacles, and resultant emotion can be detected using regular matching rules as in the example shown later in this section.
  - In case no desire (wish) was expressed in the previous few dialogue cycles (two through five, decided through fuzzy reasoning), changes among emotional words are confirmed or their related factual or emotional words/phrases/sentences in the client’s sayings (frequently occurring in dialogue session stored in the log, some with the tag such as [obstacle] or [resultant emotion]) are asked about the relationship with the desire (a word or phrase tagged by [wish] and stored in the log) as the reply.
  - If more than one of the above rules are applicable, detect the most appropriate one through fuzzy reasoning.
  - At the beginning of the reply, insert output interjections such as “Hmm”, calculating their repetition number and length.
  - Generate the reading of Japanese “kanji” for the derived output based on Kanji reading (Ruby) dictionaries.
7. Stop the conversation if there is a final positive remark by the client such as “I feel refreshed” or “It was good” or there are no further clients input for the specific duration.
8. Analyze the current and former dialogs of this client (their length and their degree of success) for output generation accordingly.

CRECA performs the rule-based analysis of input sentences by pattern matching and the creation of a response/log for paraphrasing, digging, and summarization.

*Example (CRECA dialogue)* Let us consider the following dialogue example of self-awareness:

Client’s input: For me, **software developer** is a wish in life, but lack of programming **skill** is an obstacle. Job hunting makes me feel worried.

VICA’s reply: *Hum, Hum, for you*, software development is a wish in life, but lack of programming skills is an obstacle. *Job hunting makes you feel worried, doesn’t it? Please speak a little more in detail*.

Client’s input: I am always the last student that finishes given program problems in my programming class.

VICA’s reply: *Hum, Hum, you are* always the last student that finishes given program problems in your programming class. *Please say more about it.*

Client’s input: I have no **skill** of rapid programming.

VICA’s reply:* Hum, Hum, you have* no skill of rapid programming. *Please tell me the relation among lack of programming skill and software developer.*

Client’s input: Oh, I found I can develop good quality programs and I would like to do research and development of software

VICA’s reply: *Hum, Hum, you found you can* develop good quality programs and you would like to do research development of software, wouldn’t you. *Please tell me more.*

Client’s input: Now I am fine.

Below are network-related techniques used in VICA [4]:

- AI terminal: JavaScript/Json on Google Chrome interfacing Speech Recognizer/Generator
- Speech Recognizer: Cloud Speech API (Google) on Google Cloud Platform
- Web Server: Nginx
- CRECA server or Counseling/consultant AI Portal (remote method of CRECA intelligent object/agent): (WebSocket Script +) Python.

### Problems of VICA as medical application use case on 5G/6G

However, in the current 4G networks, there are problems as follows: (1) It has an error every two sentences or so^4^. (2) Some words drop or disappear during recognition. (3) It sometimes takes more than 10 s in response time, or in the worst case, the connection/task fails altogether. Thus, in 4G networks without slicing, the cloud-native speech recognition of VICA has a reliability problem. It is a significant problem, because VICA uses “sensitive listening” skills as its counseling methodology [7]. The skill requires sensing or listening to clients’ minds through recognizing clients’ sayings and their very sensitive emotions with extreme accuracy. Then, it continues conversation or communication to promote clients’ reflection towards their self-awareness. Thus, VICA has a serious reliability problem in such speech recognition of AI cloud services. Even though accurate image recognition may help to solve the problem, accurate emotion detection/recognition from such as facial expression is further difficult due to a large amount of image data transmission burden in networks. Such reliability problems of AI recognition Cloud services have to be solved, so that our AI counselor “VICA” can be practical in real use.

It is important that VICA further guarantee or assure speech recognition accuracy/reliability in Google speech-to-text cloud service. To attain this, also using the distributed computing approach, VICA servers more densely collaborate with its clients/terminals. In this case, VICA has another problem of transmission over inter-carriers in the reliability and/or latency of networks if terminals are located far from servers as those in foreign countries. VICA has clients for speech I/O (Input/Output) and servers for AI counseling. Clients composed of PCs (Personal Computers) or mobiles handle speech/image recognition to obtain the input from patients. Servers mainly create responses for counseling from the input. However, for VICA using “sensitive listening” type counseling methods, speech recognition quality is fatal. VICA servers must assure it before creating the response for counseling. It is done as follows. (1) VICA terminals send voice data to AI speech recognition Cloud servers in such as Google Clouds. (2) VICA terminals receive the recognized speech text. (3) The text is sent to VICA servers. (4) VICA servers validate the resultant text by perplexity checking, etc. (5) On suspecting the error, they improve by several methods before CRECA generates a response for AI counseling. For example, these methods are various kinds of n-gram (forward, backward and so on) with weighted majority decision [11]. (6) If the error is still suspected, servers send text messages to ask clients (terminals) to redo the speech recognition using Google Cloud. (7) To attain high accuracy, this loop should be repeated until the error is not suspected. Meanwhile, the number of loops is limited, so that the loop stops when elapsed time becomes more than the specified time. The elapsed time is defined as *t*$_{c}$ (current time)-*t*1 (input starting time of the current dialogue). The specified time can be adjusted. However, the maximum time is also limited, since too long dialogue response time very often stops the conversation. Thus, the network performance affects the number of loops, which affects the quality of speech recognition service. Thus, an ultra-reliable low-latency network such as that of 5G with slicing is required. The next section shows these network requirements.

During emergencies, extraordinarily many persons will use counseling AI systems at the same time. For example, in large cities, around 70K possibly more than 100k persons are expected to use VICA concurrently [4]. Thus, not only reliability or latency but data rate (bandwidth, transmission speed) becomes problems during emergencies or a surge in demand.

### Requirements for VICA for medical application on 5G/6G

To solve problems or to realize expectations mentioned in the previous section, VICA requires no disconnection, less than one word every ten or more sentences of recognition error, and less than 10 s of total response time. This is indispensable for distressed/anxious persons (clients) to continue the conversation or virtual meeting via voice or further including image and other sensors towards self-awareness.

Thus, the network requirements are considered as follows:

1. Requirement for text: network delays including inter-carrier should be shorter than 100 ms, due to several times repetition of communications among their clients and servers to control the recognition error recovery and the automatic recovery processing time (e.g., several 100 s) of the server computer. A data rate of around 10–100 Mbps is required between the AI terminal and AI server when 10–100K persons are using VICA concurrently during emergencies.
2. Requirement for voice (including tone, majority decision for quality improvement): network delay should be 5–10 ms. Speech conversation interval totally permits a second. Speech recognition in cloud sometimes takes a second. It may be a problem. Thus, rather, much lower degree of a second should be necessary. Data rate should be 15–100 Gbps upstreaming to Google speech-to-text cloud especially for, e.g., 10–100K persons concurrently use during emergencies.
3. Requirement for Video (3D, 180 degrees for facial expression recognition): network delay: 1 ms for instantaneous recognition of facial expression, 500 Gbps Video upstreaming from AI terminal to cloud-based image-recognition/multi-sensor-fusion servers for only around 1K persons concurrently (8K UHD (Ultra High Density): 100 Gbps, even if highly compressed and very low quality, 0.5 Gbps)

Requirement details for delay are as follows:

Currently, experiments show that 5G is not sufficient for even just AR (Augmented Reality) or AD (Autonomous Driving). Meanwhile, Counseling is a kind of mental operation. Even one word of slow, missing, or incorrect recognition of the client’s voice can easily stop the conversation. It stops the counseling for mentally distressed persons or the consultation during an emergency. Counseling (consultant) AI requires quality communication such as less than 1 recognition error per 100 sentences or 1000 words. It is 10 times that of excellent speech recognizer’s quality. For iPhone or google assistant, it is 1 error per almost one sentence (much less than two sentences) or per 20 words or Japanese characters^4^. Even the high-quality Google speech recognizer makes speech recognition (text conversion) error of around every two sentences. It sometimes causes more than a few seconds delay on the cloud. Furthermore, there is also an encryption overhead for secure (privacy-preserving) communication that is necessary for counseling or consultation.

Consequently, in speech conversation, even the Cloud Google speech recognizer has errors.

Such requirements detail for data rate are as follows:

Counseling/consultant AI (VICA) requirement for Voice (including tone, 5 majority) is upstream over 10–20 (− 100) Gbps. For example, during the Coronavirus emergency, at least one prefecture had more than 1K counseling calls/day even before its explosion, simultaneously 1K calls maximum possibly. Each voice call requires 1.5M (=64 Kbps*24 channel) bps [13]. 64 Kbps can be compressed to 32 Kbps in no loss of sound quality and 1.5 Mbps becomes 0.75 Mbps. However, the majority decision out of 2-5 cloud speech recognitions makes it more than 1.5 Mbps again. There are also problems with surrounding sounds and echoes for long distances.

Besides AI counseling calls, there are basically Internet telephone (inclusive of human counseling or just asking information, etc.) especially after school or office. Tokyo population is 14 M. On Corona explosion, if 7–14(− 70) K persons (e.g.) expected to have such calls at once during several minutes, upstream will require (1.5 Mbps * 7M =) 10–20–100 Gbps in Tokyo. It takes much more if the canceling of noise such as echo, surrounding sound, etc. is considered as well as compression with high quality maintained even on various tones such as rising/faint tones. This is because speech recognition mistakes of even one word can stop counseling. It is still worse if the connection/task fails in the worst case.

For realizing the very accurate/stable recognition required by “sensitive listening” of VICA, the next section proposes the method to fulfill and assure these requirements, using 5G/6G slicing inclusive of Cloud.

## Basic solution to VICA as 5G/6G medical use case

This section proposes the basic solution to VICA’s problems/requirements as a medical application use case on 5G (/6G) for remote mental care to emotionally distressed persons. A high-performance distributed system and optimal cost networks inclusive of Cloud are necessary for VICA’s establishment of reliable non-disconnect speech recognition. It should be provided to cope with the surges in demand during pandemic/disasters when networks are extraordinarily congested or disconnected. The fast and stable network operation is important to initial fulfillment and dynamic real-time assurance of the requirements.

### Proposed basic solution

**Fig. 3:**
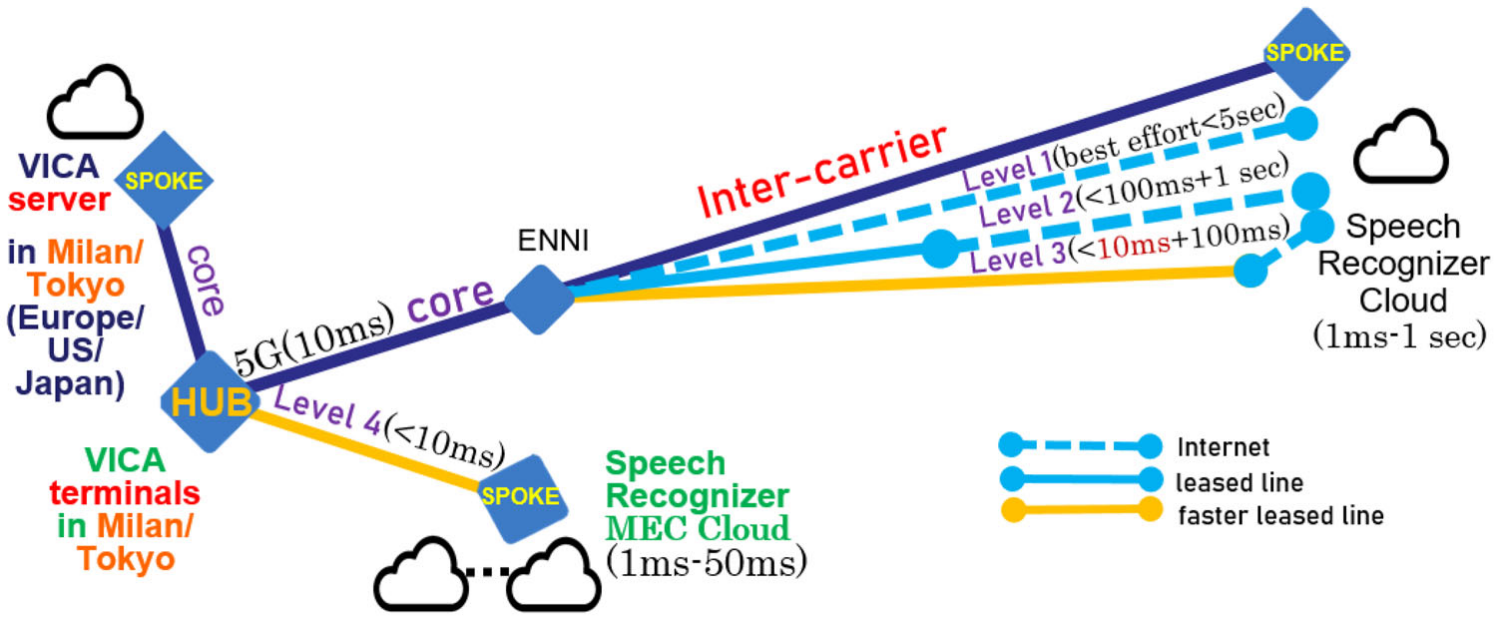
Network configuration for solving cloud-native speech recognition problems. ORQ/MRQ/HRQ/VHRQ: Ordinary/Middle/High/Very High Requirement Quality

The proposed solution is a cloud-native distributed system architecture with a service catalogue providing the multiple levels of the menu to assure the service level by such as SLA (Service Level Agreement). This stably enables high performance as well as an optimal cost network. Towards 5G/6G use case PoC of VICA, it aims at preventing cloud speech recognition from frequent errors (e.g., every short sentence) and significant response problems/delay or disconnection in the worst case. The flexible network configuration is illustrated in Fig. 3 where External Network-to-Network Interface (ENNI) is also presented. The service catalogue is proposed to cope with congestion during emergencies, disasters, etc. For instance, users at the VICA terminal can select a menu from the following 4 levels of the service catalogue:

1. level 1: ordinary: ORQ (Ordinary Requirement Quality: best effort), allocating Internet, aiming at; response time: 500 ms (possibly over 10 s) and false rate: 1 phrase block of recognition error per 1 sentence (not assured, possibly many errors) and monthly fee: $1
2. level 2: MRQ (Moderate Requirement Quality: affordable quality on congestion), allocating 5G (if usable) and ordinary leased (dedicated) line or Bandwidth guarantee VPN (Virtual Private Network), recognition error recovery included, aiming at; response time: 50 ms in average (less than 10 s assured with 99%) and false rate: 1 erroneous word per 10 sentences (not more than 2 phrase blocks of recognition error per sentence, assured with 95%) and monthly fee: $10
3. level 3: HRQ (High Requirement Quality: affordable quality on emergency), allocating 5G (if usable) and long and multiplex fast dedicated (leased) line, multiple (redundant) inter-carrier facility for improved speech recognition error recovery (described in the next section), aiming at; response time: 10 ms in average (less than 3 s assured with 99%) and false rate: 1 erroneous word per 10 sentences (not more than 1 phrase block of recognition error per 3 sentences, assured with 95%) and monthly fee: $20
4. level 4: VHRQ (Very High Requirement Quality: high quality on ultra-emergency), allocating 5G (if usable) and MEC Cloud, multiple (redundant) inter-carrier facility for elaborated speech recognition error recovery (described in the next section), aiming at; response time: 5 ms in average (less than 3 s assured with 99.9%) and false rate: 1 erroneous word per 30 sentences (not more than 1 phrase block of recognition error per 10 sentences, assured with 95%) and monthly fee: $50

In such a model, the service-level catalogue for quality counseling needs to be more than level 2 to maintain speech recognition quality. The cost is paid by health insurance, hospitals, or the user’s subscription fee. In an emergency, ministers can order automatic 1–2 level upgrades of catalogues, whose cost is paid by the national tax. Such catalogue is shown by VICA terminals. Users (clients or patients) select the service level (1–4) in the catalogue menu. VICA terminals send it to AI counselor server (VICA server). VICA server reports to manage it as a basic requirement to EA (Enterprise Application). EA reports the basic requirement to CRM (Customer Resource Manager). Through the report, NaaS (Network as a Service) via BSS (Business Support System) of CSP (Communication Service Provider), etc. assigns network/Cloud resources including MEC (Fig. 3) to fulfill the basic requirement according to an Service Level Agreement (SLA) and the like.^5^ Meanwhile, the VICA terminal monitors the delay and sends it to the VICA server. VICA server logs the overall delaying situation of each service level to analyze the problems for VICA 5G use case PoC. VICA server reports it also to EA /CRM. As shown in Sect. 4.2, through the report, NaaS via BSS can perform optimal network operation to assure the basic requirement.

### Quality assurance operation

This section proposes the assurance method for VICA 5G use case PoC. Assurance/re-fulfillment operation is important for dynamically solving the above-mentioned VICA’s problem in the AI speech recognition cloud.

As described in the previous session, the base requirements of the service-level catalogue are initially selected by users (clients), set by AI counselor terminals (VICA terminals), and fulfilled by NaaS of CSP, etc. However, during counseling sessions also, they are kept assured dynamically by NaaS as follows: AI counselor terminals (VICA terminals) monitor the delay during a counseling session and send it to AI counselor servers (VICA servers). VICA servers dynamically report the overall delaying situation of each emergent level to OSS (Operation Support Systems)/BSS of NaaS. It is done by network operation API such as Skynet in the EA domain. On the detection of serious delay, NaaS automatically performs the network operation such as slicing to assure or, in case of changing the network configuration re-fulfill the basic requirement dynamically in real-time.

The next section proposes a way to further improve the reliability of VICA using AI recognition cloud service inclusive of AI speech recognition as well as AI facial expression detection. To implement such a solution for improving speech recognition accuracy a counseling agent VICA is extended as advanced VICA in its distributed system architecture. It also proposes a way to estimate the effect quantitatively, especially for 5G use case PoC. This helps to show the usefulness of 5G to VICA and its problems causing the necessity of 6G.

## Enhanced/advanced VICA as 5G/6G medical use case PoC

This section describes the enhanced VICA and advanced VICA. VICA has been developed for accurate as well as stable recognition towards more reliable “sensitive listening”. The quality assurance method is described including networks.

First, in the enhanced version, the VICA server validates and corrects the speech recognition result (the erroneous text) which the VICA client obtained using Google’s AI speech recognition clouds. Though communicated with text, the VICA server may be connected over inter-carrier networks. The VICA enhanced version is expected to make use of 5G and slice over inter-carrier networks.

Second, the subtle changes in clients’ emotions are difficult to detect in just speech. They often do not say it or say it vaguely, which mostly appears in their body language such as body movement, facial expression, heart rate, blood pressure, even oxygen level, etc. Advanced VICA tries to understand body language (first facial expression) besides natural language (speech). Body language understanding is done by multiple sensor fusion, though the results are the text or sign indicating the feeling, such as negative, neutral, or just a bit more positive, and positive. If they utter their emotion by speech directly, advanced VICA can integrate it through the emotional word dictionary. Such integration enables ultra-reliable “sensitive listening”. This is indispensable for VICA. Emotionally distressed or anxious persons can continue the empathetic less-intrusive confession-like conversation. Their emotional change detected by utterance (speech) or body language (facial expression) is notified by VICA. This promotes their reflection towards self-awareness. Advanced VICA (advanced version of VICA) makes full use of 5G/6G networks. They provide ultra-reliable, low-latency, high-speed network services along with edge-enabled servers (MEC: Mobile/Multiple Edge Computing). Using 5G/6G networks, real-time/recorded video and other sensory information are processed together with speech information.

To realize such ultra-reliability by 5G/6G, VICA including the enhanced/ advanced version has the performance (response delay) monitor and enables the 5G/slicing operation including the change of network configuration. Section 5.1 describes the enhanced VICA approach towards more reliable speech recognition. Section 5.2 clarifies the implementation more concretely. Section 5.3 describes the performance improvement method of the advanced VICA including image/sensor data recognition and analysis.

### Enhanced VICA as 5G/6G use case to improve speech recognition quality

Network resource fulfillment is done according to the basic requirements as mentioned in the previous section. Namely, network configuration or slicing is set initially when users selected the service level (catalogue menu) of the AI counseling application VICA. Depending on the social situation including the number of patients and the minister’s public requests on the pandemic, the slicing (network resource allocation) is done dynamically and automatically. It can be done proactively beforehand though possibly in 5G/6G or in the future. This is because it takes too much time to finish such slicing especially when it is done manually. More dynamically, the AI terminal of VICA monitors the delay in real time and sends it to the AI server of VICA. The VICA server at the EA domain reports the overall delaying situation of each emergent level to service providers (CSPs). Through the delaying information, NaaS of CSP can change the network resource or its configuration to assure the basic requirement via CRM and BSS/OSS.

Furthermore, at this point, 5G facilities are limited or very expensive. Both 5G and automatic network operations are not available for our experiments. To cope with this problem, our 5G validation experiments for 5G use case PoC of VICA simulate the effect of network (slice) operation by substituting the cloud (remote) server with the local server.

**Fig. 4:**
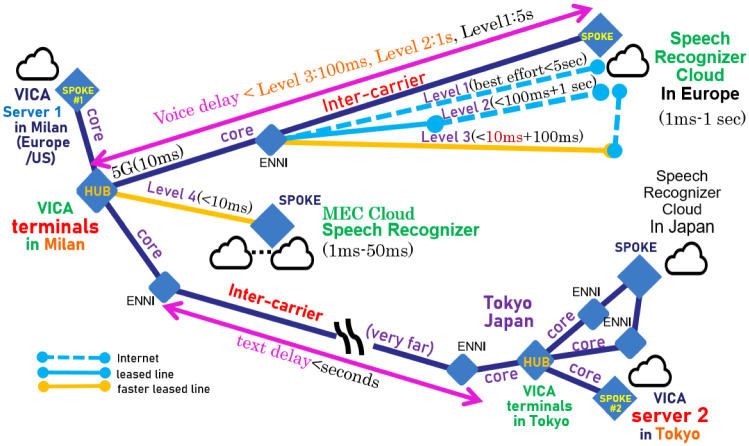
Enhanced VICA network configuration for improving speech recognition cloud

Figure 4 shows this problem and solution approach for 5G PoC of VICA use case as follows: Problem: At this moment, not 5G but 4G is used in a city in Japan. Even text transmission delay among VICA client (VICA terminal) and VICA server significantly decrease the effect of 5G (not including transport slice) use if the AI server (VICA server) is set in the city in Japan. Approach: AI server is set also in European/American communication provider’s edge or core network to simulate 5G slice effect of less latency in edge compared with less latency effect in inter-carrier.

Method: Emergency base prioritized implicit network operation effect for 5G (shown by delay data at AI terminal) to Google Speech Recognition Cloud as well as to AI counselor server 1 set in Europe/US is compared with the effect to AI counselor VICA server 2 set in a big city in Japan. Just VICA server 1 in Europe or the US sends initial base requirement of emergency level (catalogue menu) selection and dynamically (during counseling) monitored response time to CRM/NaaS through network operation API such as Skynet for 5G slicing.

### Evaluation method for 5G/6G use case PoC of enhanced VICA

**Fig. 5:**
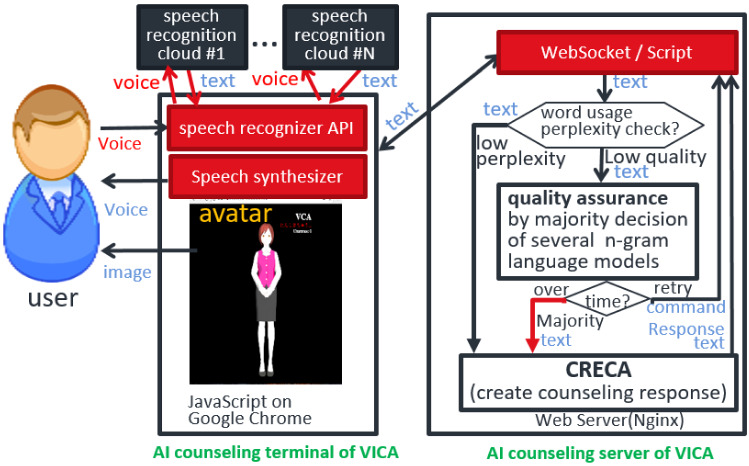
System architecture of enhanced VICA

This section describes the network architecture and evaluation method for 5G/6G use case PoC of enhanced VICA. Figure 5 shows the architecture of enhanced VICA. Using AI speech recognition cloud, AI counseling Terminal (VICA terminal) obtains text as the speech recognition result. However, in the showcased use case scenario. the text is erroneous or missing as mentioned before.

Communicating with VICA client, AI counseling server (VICA server) collaboratively improve the result as follows [11]:

1. Perplexity check is done for the resultant text received from the VICA terminal.
2. Automatic correction is triggered if errors are suspected due to perplexity indicators.
3. Automatic correction of AI speech recognition results is executed by various kinds of n-gram (forward, backward, etc).
4. Majority decision is done or speech recognizers are activated again if the results are different from each other and have low-quality perplexity indicators.

**Fig. 6:**
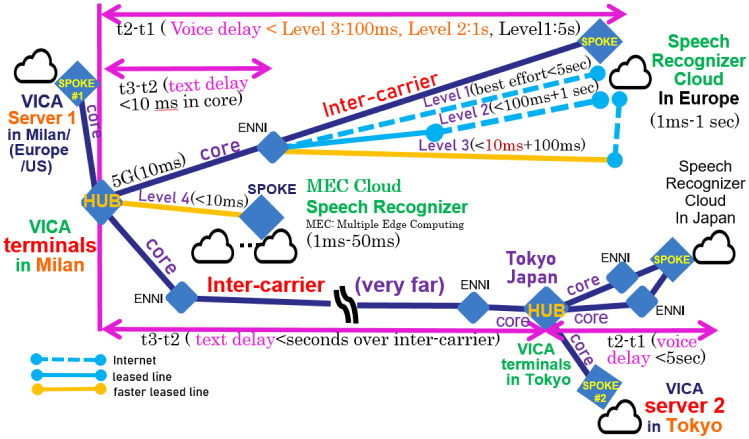
Network configuration of enhanced VICA for speech recognition reliability improvement and its evaluation

Nowadays, people in foreign countries need counselors in their mother country. In such cases, the server of VICA is connected with VICA terminals over inter-carrier networks such as between Europe/the US and Japan. In the case of the VICA server collaborating with VICA terminals for improving speech recognition reliability, the communication is repeated many times if errors continue. In an emergency, such communication over inter-carrier networks increases sharply. To decrease the delay or burden over inter-carrier networks in such cases, VICA enhanced versions are expected to make use of 5G/slice.

For the 5G/6G use case, PoC of VICA, network operation, and its validation to improve cloud speech recognition reliability are described in two cases of VICA server deployment. For instance, as Fig. 6 shows, VICA server1 is deployed in Milan in Europe and VICA server2 is in Japan. In these two cases of deployment, VICA enhanced version performs quality assurance through network operation API for 5G/slice to decrease the burden over inter-carrier as follows:

1. Initially, VICA terminals in Europe send base requirement of users and monitoring results to VICA server as follows:
  1. VICA terminal shows the service catalogue to users (patients) and sends the selection as a base requirement.
  2. VICA terminal monitors such as Voice input starting time *t*1, Speech recognition completion (on event handler starting) time *t*2, and AI counselor’s speech output time *t*3 at each dialogue of VICA. VICA terminal sends the monitoring result of *t*1, *t*2, *t*3 to the VICA server.
2. VICA server initially sends the base requirement from VICA terminals. Then, during counseling, it sends the monitored delay results also from VICA terminals. Both (base requirement and monitored delay results) are sent to CRM and NaaS CSP (service providers) through network operation API such as Skynet. Server1 (in Europe/US), as well as server 2 (in Japan), connected over the inter-carrier log the monitored delay data. They are comparatively analyzed and evaluated in two cases of deployment for 5G PoC as follows:
  1. VICA server in Europe or US sends the base requirement according to emergency level 1-4. It also sends the monitored duration (*t*2 – *t*1) or (*t*3– *t*1) for each conversation at each level.
  2. VICA servers both in Japan and in other countries such as Europe or the US log the base requirement (ordinary level, emergency level, etc.). They also log the monitoring result *t*1, *t*2, *t*3 to each requirement level for estimating the assurance in the 5G slice. The time *t*3 – *t*2 for the VICA terminal in Europe/US to access the VICA server in Japan over inter-carrier can be over 10 s in an emergency.

### Advanced VICA fusing sensor/image for accurate recognition and its assurance method

Emotionally distressed persons/clients do not verbalize their emotions very much. Even if they say it, there is a good chance that their utterance becomes faint or unclear. Sometimes, even humans cannot catch their voice. In addition, speech recognizers may fail to recognize the voice. Especially, for “sensitive listening” type counseling advocated by Rogers [7] which VICA adopts, it is important to know clients’ emotions or their change very accurately without intrusion or without asking them explicitly. Human counselors know client’s emotions through their facial expressions or physical behavior. Meanwhile, advanced VICA tries to do this through the fusion of sensor/image data, incorporating real-time closed-loop feedback. To produce emotional words that VICA uses for the creation of counseling responses, the result is integrated with one obtained from the speech recognition of the user utterance.

Facial expression recognition is used to evaluate facial cues to interpret and understand the emotional state of patients, including emotions such as anger, fear, happy, or sad to assist in providing proper assistance. As to this, we have the first version of facial expression detection for mental stress. Sad feelings to indicate anxiety and happy feelings to indicate “clear up” or “fine” (“solved” or “self-aware”) are detected though a bit too simplified as the beginning attempt. Defining the former as a negative feeling and the latter as a positive feeling, for example, the change is detected in the feeling during the consultation. The change of emotion is very important for VICA to let patients reflect towards self-awareness by saying “You initially said xxx but now you are saying yyy”. Facial expression detection AI integrated into VICA can learn automatically as well as incrementally by logging the pairs of words (user utterances) and the corresponding facial expression. Using such incrementally learnt/trained face expression detection AI, VICA can recognize emotional change even if patients do not say any emotion words or say them unclearly [14–17]. If patients do say emotional words, the accuracy of such emotional change recognition is improved by majority decision, etc. As to the improvement by majority decision, extracting continuous facial data to analyze essential features captures vital health signs such as stress level, fitness level, blood temperature, and blood pressure. Integrating this information also, advanced VICA can have another way of emotion recognition in near real time, which contributes to more accurate emotion change detection by majority decision. With the sophisticated algorithm and cloud-nature supreme processing power, the possibility is limitless. These improve the performance (total accuracy or reliability) of VICA.

Recognized emotion can have more degrees such as very sad (very negative), neutral (neither sad nor happy) or a bit happy, and very happy (very negative). Anxiety degree for advanced VICA is defined as such in terms of facial expression measurement. It is integrated with emotion detection by voice/speech recognition. This allows it to make near-real-time judgments and selection of words for communication with the patient. Thus, whole expression logic is integrated with speech recognition. This collaboration is indispensable to enhance the total accuracy of emotion change recognition for counseling or consultancy providing vital health advice as required by “sensitive listening” type VICA.

The advanced solution for a complex scenario where a patient seems smiling, but is still under stress will be implemented soon (e.g., movement of lips, movement of eyebrows, movement of the chin, movement of chicks, eyes movement, dialogue delivery, head movement, heart rate, blood pressure, and oxygen level). Of course, this will be combined with analysis of sound output, dialogue delivery, etc. as part of the audio analysis.

Eventually, combining all such recognition/analysis of various/large volumes of data, we can realize medical grade or very accurate sensor fusion necessary for AI counseling. Due to such ultra-reliable high-performance transmission/analysis of large data, VICA is further considered as use case of 5G/6G.

**Fig. 7:**
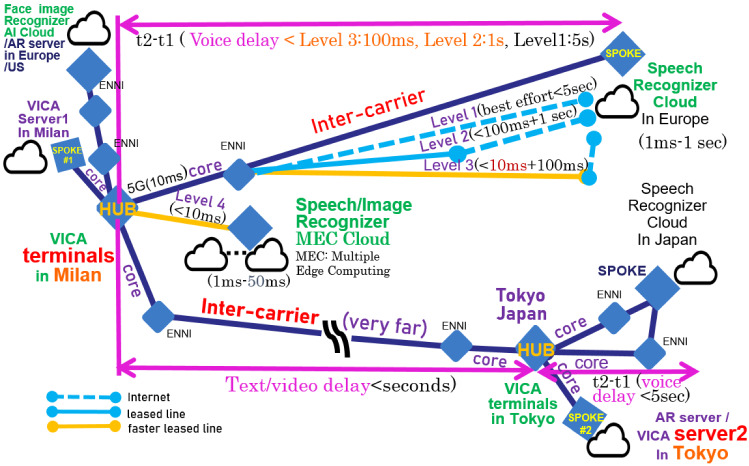
Network configuration of advanced VICA and AR server for remote human counselor

As shown in Fig. 7, for 5G/6G use case PoC of VICA, we propose two types of server deployment for counseling. The one is an advanced VICA equipped with AI servers for sensor/image fusion. The other is the human cooperation version of VICA equipped with AR servers for remote human counselors cooperation. These include image data transmission as follows:

Full AI counselor/consultant: body language including facial expression is recognized by AI

1. Clients’ body language such as facial expression, head movement, etc. is recognized by image-recognition AI (/emotion detection) cloud (server).
2. Clients’ physical condition such as pulse, body temperature, etc. is fused by AI clouds (servers) for emotion detection.
3. These AI clouds (servers) are set in Europe/US communication provider’s core networks or edges where VICA terminals are located. VICA terminals measure *t*1 at voice input, *t*2 at recognition (on event handling) completion, and *t*3 at voice output.
4. VICA terminals send the monitor results *t*1, *t*2, *t*3 to the VICA server, so that the server can check and recover/improve the recognition results on the suspicion of errors. This improving loop is repeated until the error suspicion degree (measured by perplexity) becomes less than specified criteria or (current time (*t*$_\text {c}$) - *t*1) becomes more than specified time.
5. VICA servers send the base requirement and the monitor result through network operation API such as Skynet.
  1. VICA servers in vicinity send base requirement and monitored delay (*t*2 – *t*1) or (*t*3 – *t*1).
  2. Both types of VICA servers log the base requirement and monitored results *t*1, *t*2, *t*3.

AR servers are set independently as well as remotely for remote human counseling.

**Fig. 8:**
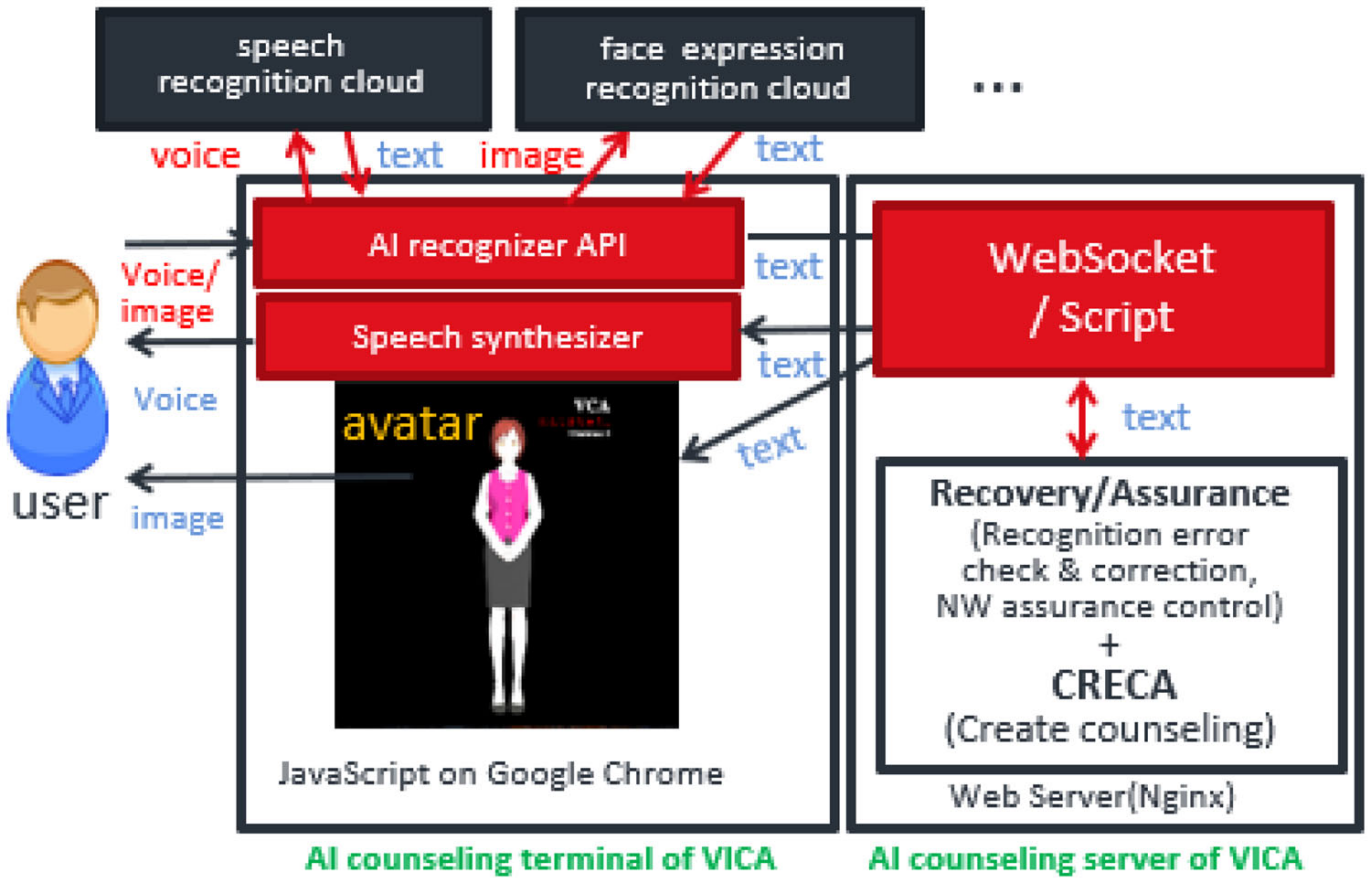
VICA advanced version system architecture

The system architecture of the VICA advanced version is as shown in Fig. 8. Compared with VICA basic version, besides voice data, a terminal of the VICA advanced version obtains various kinds of sensor data such as facial expression images from emotionally distressed persons. It sends them to the cloud-native AI recognition servers for analysis. It receives textual signs indicating the client’s emotion or condition. These textual signs and client’s utterances are sent to the server of the VICA advanced version. The server of the VICA advanced version (VICA server) integrates/fuses this information. If errors are suspected, such recognition (including speech) and integration are repeated for ultra-reliable (very accurate and stable) “sensitive listening”.

## Evaluation results

This section describes the quantitative and qualitative evaluation of VICA (basic version) on 4G to unveil problems on conventional networks and clarify the necessity for 5G/6G.

### Quantitative evaluation by experiment and its analysis

Cases including congestion-period, 4G, local server, and no slice were measured for experimental evaluation of VICA. More specifically, the conditions were as follows: congestion period (4 pm-11 pm), 4G and no slice from AI terminal to Google cloud (AI terminal is in Tokyo Metropolitan Area), but ultra-speed network simulating 5G/6G connection among AI sever and AI terminal by setting local AI server.

Results are shown in Tables 1 and 2 and can be interpreted as follows: For the AI terminal, the experiment utilized Windows 10 Home machine having Intel Core i7-5500 U CPU, 4 GB RAM, and 453 GB HDD, while for the AI server, Windows 10 Pro machine having Intel Core i7-7700 CPU, 32 GB RAM, and 953 GB HDD was used. Four Japanese nationals were used as human test subjects: a 30 year old female IT worker, a 50 year old male IT chief engineer, a 50 year old male retiree, and a 70 year old IT scholar. They were given the paper containing Japanese sentences to speak to VICA for counseling and the paper to write the test results. The contents of the sentences are shown in Table 4. The length/number of the interchange (dialogue/conversation) is ten. Each interchange has 1–2 sentences. They talked to VICA according to the given sentences. The test was repeated ten times per subject. Subjects recorded the number of VICA’s speech recognition errors (wrong recognition of each word) as well as disconnection or no response from VICA.

As shown in Table 1, using 4G networks during the congestion period, even just speech recognition time *t*2 – *t*1 was more than 5 s, which occurred three times out of seven times. The total response time *t*3– *t*1 for one dialogue was more than 10 s in 2 of 7 cases. These never satisfy the service requirement for response time as is described in Sect. 4.

As shown in Table 2, VICA (basic version) sometimes failed in Google cloud speech-to-text service. It happened in case of unclear pronunciation or stumbled/clogged wording. For various persons, it did not happen in the local service of Google speech-to-text without using the network, though it is not dialogue/conversation just commanding or dictation. Indeed, in this case, the speech recognition error occurred but complete failure did not. Meanwhile, a speech recognition error in dialogue that sometimes can stop conversation continuity occurred. It is fatal in counseling. The counseling server communicating with the client should detect and recover it semantically before creating the response.

As shown in Table 3, speech recognition errors in Clouds became more than double when compared with those in Edge. (Namely, Clouds 25 versus Edge 12 in dropped/disappeared sentences or phrases during recognition and Clouds 38 versus Edge 10 in recognition error to wrong words). Here, Google speech-to-text Clouds was used as the Cloud, and speech recognizer of Google Pixel 3A mobile was used as the Edge. The results were obtained using 11 Japanese sentences that are difficult to recognize. These examples are shown in Table 4. These example sentences are repeated in three kinds of speed such as normal, a bit slow, and slow. VICA, using the Cloud, had too many errors in the very fast utterance of such long sentences to continue the experiment. However, the Edge could recognize.

These results indicate that the proposed catalogue-based quality assurance mechanism using 5G/6G slice inclusive of MEC is necessary as well as expected useful for VICA. The reason is clarified as follows:

1. Speech recognition errors in the case of using the edge were less than half when compared with those using Internet clouds. The edge computing did not cause the failure which can be fatal to counseling due to the conversation halt.
2. Catalogue of high level (e.g., emergent level) enables agile automatic creation/selection of slice using MEC (in case of emergent level). Due to this slicing, VICA users choosing the emergent level catalogue can use edge computing (MEC) when speech recognition quality is very high and few failing down occurs.
3. Catalogue of low level (e.g., ordinary level) uses Internet which is the best effort and does not assure the performance. Indeed, the setting is possible to use MEC slice dynamically by real-time delay monitoring. However, it is far from quality assurance, since the low priority of MEC slice usage is assigned to low-level catalogue selection users. Especially in an emergency, due to a shortage of resources, this low priority makes it difficult in agile switch to the MEC slice, which causes recognition errors and occasional connection failure.
4. On the contrary, resources or readymade MEC slice can be prepared in advance to some extent for regular customers subscribing to high-level catalogues. High-level catalogue customers are provided agile usage of high-performance 5G/6G slice even on emergency access.
5. Thus, the multi-level catalogue using a 5G/6G slice is necessary and effective.

**Table 1:** Measured time (speech-to-text clouds, congestion period: 4 p.m.–11 p.m.)

Id	DayOfTheWeek&Time *t*1 (ms)	Voice *t*2 – *t*1 (ms)	CRECA *t*3 – *t*2 (ms)	Total *t*3 – *t*1 (s)	Error(words/sentence)
1	Sunday 19:03	22209	2050	24259	0/1
2	Sunday 19:05	3766	2057	5823	0/2
3	Sunday 19:05	4908	2032	6940	0/3
4	Sunday 19:07	3970	2039	6009	0/4
5	Sunday 19:07	6769	2044	8813	1/5: 20%
6	Sunday 19:08	4040	2054	6094	1/6: 17%
7	Sunday 19:08	10978	2028	13006	1/7: 15%

**Table 2:** Measured time in milliseconds (ms) for speech recognition/total and word recognition errors in dialogue sentences (Underline shows translation error. “Too big” shows “unmeasurable” due to disconnection.)

DayWeek&Time	Voice	Total	User’s saying
Thursday 20:57	Too big	Too big	Failure of cloud speech to text due to stumbled/clogged word
Thursday 20:58	3434	7018	
Thursday 20:58	5325	7748	
Thursday 20:59	Too big	Too big	Fall of cloud speech to text due to unclear pronunciation
Thursday 20:59	10108	12507	
			(word errors: long sentence)

**Table 3:** Speech recognition errors (Clouds versus Edge)

Id	DayOfTheWeek&Time TalkSpeed	Disappear	Disappear (phrase)	Disappear (word)	Error (wrong word)
1	Sun 11:00 normal	8	25	38	13
2	Sun 11:10 normal	1	1	2	4
3	Sun 11:20 semi-slow	7	14	20	12
4	Sun 11:30 semi-slow	5	23	40	2
5	Sun 11:40 slow	10	25	43	13
6	Sun 11:50 slow	6	7	10	4
7	Cloud.vs.Edge	25:12	64:31	101:52	38:10

**Table 4:** Dialogue sentences used for speech recognition reliability measurement

ID	User’s saying
1	
	(I’m having a hard time writing my thesis, I’m worried that I’ll be late for the deadline.)
2	
	(I’m behind in writing the thesis, I’m worried that I’ll be late for the deadline.)
3	
	(Even when I search the literature, I get tired of my eyes on the Web..., and if this continues, my eyes hurt...)
...	.........
N	
	(Well, We don’t have fever, so I can afford to believe both of them are not infected with Corona. Oh, I have solved it, but
	still the immunity ...)

### Application quality evaluation through questionnaires

It causes a serious negative impact when clients become too irritated to continue counseling especially in case of emergencies on pandemics such as COVID-19. Thus, the final user effects on our use case VICA were evaluated mostly during the network busy period. Namely, the following user-level (i.e., application level) questions were posed to five distressed persons/patients (clients):

1. Were you irritated with delayed response inclusive of speech timing in Web/virtual conversation or meetings for counseling?
2. Were you irritated in voice conversation quality inclusive of recognition error and output voice quality?
3. Did you have stressful experiences of heavy response on connection inclusive of speech recognition stop errors causing conversation discontinuity?
4. Did you feel or experience a general but significant inconvenience such as output voice quality?
5. Did you feel high-level emergency catalogues incur high expense due to such as MEC (Multiple Edge Cloud or backbone/core network cloud) or a leased line to/near the cloud center?

Each item has the following selections: Type A. problems; Type B. small problems; Type C. no problems. As shown in Table 4, the results were as follows:

1. Even on domestic usage in Japan under 4G, 80 % persons indicated type A problems caused by network delay (concretely on network-related failure combined with speech timing) and 60 % persons indicated type B, 40 % indicated type A problem of irritation in speech-to-text translation inaccuracy.
2. In one of these experiments, a client lives in the suburban area and used Amazon’s AWS (Amazon Web Services) cloud for AI counseling service of VICA, where cloud performance (1 GB memory), as well as the communication channel (4G), is relatively poor. He indicated type A problems for all of the question items 1–4.
3. The high-cost problem was C, as the base is not expensive.

Thus, we had 80 % of the subjects who felt the problem by network delay inclusive of the network-related failure especially depending on speech timing as well as by speech-to-text translation inaccuracy in 4G. Furthermore, merely unclear voice caused the disconnection with speech-to-text cloud servers as Table 2 also showed. The word errors when saying long sentences easily happened even when speech-to-text cloud servers were disconnected. These also irritated subjects. These qualitative evaluation results showed that 5G/6G including slice is necessary.

## Related work and comparative analysis

As related works, many kinds of conversational systems such as those called chat bots have been developed [18]. Besides stand-alone conversational or consultation systems independent of other services, they include in-app assistant [19], query engine [20, 21], information retriever [22], conversational IoT (devices such as physical robots) interface [23, 24], GUI agent [25–27], business process interface [28], and API caller [29–31]. The in-app assistant chat bot lives in application software. Supe-Agent for online shopping is its well-known instance. A query engine chat bot provides conversational access to rather structured data/ knowledge base and to inspect specific instances (data) by translating natural language instructions into low-level query languages, such as SQL or SPARQL. Meanwhile, an information retriever chat bot enables natural language queries over a typically unstructured set of documents or data. They give users answers or actions/services. They are partly in practical use, though building robust intelligent chat bots such as VICA is still a challenging endeavor. Really, voice commanding systems or voice text translation services including contextually limited guidance robots [1] are somewhat in the trial use. Currently, they are distributed using such as three-tier architecture [32] and their patterns are reported to integrate [33].

In speech conversation, even high quality/response Cloud Google speech recognizer sometimes has recognition error as well as several seconds of response time on crowded network/computer resources. This can irritate even ordinary users. Google’s edge computing speech recognizer can constantly respond in the order of millisecond (ms) order. They say its users enjoy ordinary voice conversation, but it is mostly for voice commands^6^. Furthermore, this speech recognizer can work just on Google’s Pixel mobile and even such an excellent one has still sometimes had recognition error of one every several sentences ^4^. This prevents the counseling AI or emergent consultation from practical use.

Meanwhile, mental health care AI bot VICA has to be much more robust and intelligent. Providing answers is not sufficient. Basically, it should let clients or patients obtain deep self-awareness by continuing conversation. Thus, it requires aforementioned “sensitive listening” ability, that is, high-quality speech recognition or sensing capability, and least mentally distressed persons should become irritated due to recognition error or failure. As described in Sect. 3.1, for users to obtain deep awareness, digging and summarization are also necessary. They are agents’ own creating responses for empathy [34] and beyond it, different from Eliza-like simple mirroring. For instance, beyond empathy, it is further necessary for counselors or counseling agents to ask clients the relation between the words they talk repetitively and the meaningful words they used for expressing their wishes or problems. It is further necessary to capture the client’s emotion or its change such as negative feeling to positive feeling from the spoken words translated to text. It enables “sensitive listening” promoting empathy needed for the continuation of dialogue between counseling agents and emotionally distressed persons. Just mirroring or paraphrasing such as one from input voice to output response is not sufficient for Roger’s type counseling. Speech understanding is still necessary. Thus, a text-based counseling agent after translating voice to text by speech recognition servers is considered more effective than a voice-based somewhat mirroring type counseling agent. These results cause multiple or repetitive uses of high-quality cloud-based speech recognition servers along with many kinds of error recovery processing in counseling AI servers. It requires less than milliseconds of network delay among AI terminals and cloud centers as well as between AI terminals and AI servers besides multiple or repetitive use time (often 100 milliseconds each) [11] of speech recognition clouds used from counseling AI terminals and error recovery processing in counseling AI servers.

For dynamic slicing to avoid such delay, our method uses a typical approach. Namely, VICA monitors the network delay or the response time. The results including their integrals are sent to NaaS in real-time^7^. However, VICA takes more than 10 min for network resource reallocation to be effective even if it is automatically done by this kind of software-designed slicing approach in case of the sudden surge in VICA’s counseling demand on an emergency such as a disaster or pandemic. As the result, the slice cannot be in time for the sharp drop in network performance. To cope with this, VICA’s catalogue approach can select a more proactive approach using social intelligence concerning such as disaster and pandemic^8^. It can allocate more network resources in time for even the sharp drop of network performance also, though its reliability depends on that of the social intelligence as well as the quantity and quality of marginal resources.

As to the related medical (mental) healthcare use cases on 5G, a 5G cognitive system (5G-Csys) for healthcare is proposed [10]. 5G-Csys has a platform for speech emotion recognition, which can recognize users’ speech emotion. They use cloudlets (edge servers) for speech emotion recognition to decrease the latency by networks for voice data transmission and computation for emotion recognition. However, it still has a problem of the (speech recognition) algorithm accuracy with lower time complexity (little delay) for the intelligent algorithm that the data cognitive engine currently utilizes. They neither propose these solutions nor evaluate them. Meanwhile, we have proposed catalogue-based quality assurance mechanism using a 5G/6G slice inclusive of MEC and evaluated the effect on speech recognition reliability improvement by comparing the errors in Clouds with those in Edge.

## Conclusion

Despite using the Google speech-to-text cloud, VICA and its IoT robot have critical speech recognition issues in the current 4G networks. These include word dropping, delayed response, and the occasional connection fails. Thus, VICA caused significant quality problems of conversation discontinuity despite promising voice communication interfaces. We attempted to solve this problem by leveraging a 5G/6G slice inclusive of MEC and devising an enhanced VICA containing collaborating servers with the aim of increasing speech recognition reliability. However, issues such as network latency among inter-carriers and overwhelming data rate during emergency situations emerged. Hence, for better quality communication with clients and for recognizing facial expressions, advanced VICA has been designed. We proposed a quality assurance mechanism based on multiple levels of catalog using a 5G/6G slice inclusive of MEC. Our experiments revealed that the number of speech recognition errors of Internet Cloud was more than double when compared with that of edge computing. Such results show that quality assurance using 5G/6G is necessary and has increased efficiency when used with VICA Counseling (ro)bot. Image recognition AI or sensor fusion AI could be crucial for more accurate recognition of patients’ feelings exhibited by body language such as facial expressions. In the near future, experiments for video data transportation will be conducted. These experiments will aim to analyze and unveil the effects/problems of 5G for quality counseling bot/robot, as well as to improve 5G towards future network standards such as 6G.

## Data Availability

Not applicable. **Code availability** Not applicable.

## Footnotes

1 M. Kapko, 2020. 6G Concepts Gain Backing Amid Political Smacking https://www.sdxcentral.com/articles/news/6g-concepts-gain-backing-amid-political-smacking/2020/06/.

2 SDx Use Cases, 2016. Software Defined Everything (SDx) Series: Software Defined Everything Part 5: SDx Use Cases, https://www.sdxcentral.com/cloud/definitions/software-defined-everything-part-5-sdx-use-cases/.

3 Skynet Catalyst - Whitepaper v1.3, 2019. Skynet Catalyst Borderless Remote Health Care: A Reality with 5G, https://www.tmforum.org/wp-content/uploads/2019/07/Skynet-Catalyst-Whitepaper-v1.3.pdf.

4 lets-hack.tech, 2019. Recognition test of three kinds of voice inputting application and summary of the blog writing method (in Japanese), https://lets-hack.tech/review/voice-control/.

5 We note that, even today, automatic slice optimization requires almost 15 s.

6 D. Coldewey, 2019. Google’s new voice recognition system works instantly and offline (if you have a pixel) https://jp.techcrunch.com/2019/03/13/2019-03-12-googles-new-voice-recognition-system-works-instantly-and-offline-if-you-have-a-pixel/.

7 Skynet Catalyst-Whitepaper v1.3, 2019. Skynet Catalyst Borderless Remote Health Care: A Reality with 5G, https://www.tmforum.org/wp-content/uploads/2019/07/Skynet-Catalyst-Whitepaper-v1.3.pdf.

8 Network Assurance: Your Key to Proactive Operations, 2018, retrieved on September 29, 2020, https://www.cisco.com/c/en/us/products/collateral/data-center-analytics/network-assurance-engine/technology-overview-c17-740236.pdf.
